# Characteristics of unilateral tibial plateau fractures among adult patients hospitalized at an orthopaedic trauma centre in China

**DOI:** 10.1038/srep40647

**Published:** 2017-01-11

**Authors:** Yong Liu, Zhengwen Liao, Lei Shang, Wenhua Huang, Dawei Zhang, Guoxian Pei

**Affiliations:** 1 Department of Orthopaedics, First Affiliated Hospital to the Fourth Military Medical University, No. 127 West Changle Road, Xi’an, Shaanxi, 710032, P. R. China; 2 Department of Orthopaedics, First Hospital of Shijiazhuang, No. 36 Fanxi Road, Shijiazhuang, Hebei, 050011, P. R. China; 3 Department of Human Anatomy, School of Basic Medical Sciences, Guangxi Medical University, No. 22 Shuangyong Road, Nanning, Guangxi, 530021, P. R. China; 4 Department of Health Statistics, Faculty of Preventive Medicine, Fourth Military Medical University, No. 169 West Changle Road, Xi’an, Shaanxi, 710032, P. R. China; 5 Institute of Clinical Anatomy, School of Basic Medicine Sciences, Southern Medical University, No. 1023-1063 South Shatai Road, Guangzhou, Guangdong, 510515, P. R. China

## Abstract

The aim of this study was to investigate the characteristics of unilateral tibial plateau fractures among hospitalized adult patients in Xijing Hospital, to evaluate the accuracy of Schatzker classification system and AO/OTA classification system to tibial plateau fractures. We retrospectively analysed clinical data on 274 patients admitted to Xijing Hospital between September 2006 and August 2015. The patients’ demographic characteristics, admission periods and seasons, external causes and fracture types were recorded and summarized. Then the characteristics of tibial plateau fractures and the accuracy rate of these two classification systems were analysed. Schatzker type II fractures and AO/OTA type 41-B3 fractures were the most common types. The external causes differed between genders, types of employment, urban-rural residents and both two systems. In addition, some fractures were difficult to classify using Schatzker or AO/OTA classification system. Rural male physical labourers aged between 30–59 years-old were most likely to suffer from unilateral tibial plateau fractures, due to traffic accidents, falls and indoor activity injuries, or falls from height. We should pay more attention to the related people and professions, which contributed to the high occurrence of tibial plateau fractures. Besides that, further improvements are required for both Schatzker and AO/OTA classification systems.

Tibial plateau fractures, a type of intra-articular fracture in which the fracture lines involve the proximal end of the tibia, represent 1–2% of all fractures^1^,^2^,^3^ and approximately 8%^2^ of fractures in the elderly. Tibial plateau fractures are common among patients treated at orthopaedic trauma units, and as well as requiring significant medical resources, can place great economic burden on patients and their families. While, the main etiologic factors, types and epidemiological features of tibial plateau fractures are reported to vary over time and between countries, and are influenced by the geographical area and socio-economic status of the population^3^,^4^,^5^,^6^.

In previous studies, Albuquerque *et al*.^5^ showed traffic accident injuries were the most common external cause of tibial plateau fractures in Brazil (52.3%), followed by falls from height (40.2%). Elsoe *et al*.^4^ reported that falls and indoor activity injuries, traffic accidents, and falls from height accounted for 43.7%, 36.0%, and 20.3%, respectively, of tibial plateau fractures in Denmark. Luo *et al*.^6^ demonstrated traffic accidents (75.3%) and falls from height (23.4%) were the most common external causes of tibial plateau fractures in Shanghai, China. Therefore, the major external causes of injury in Denmark (falls and indoor activities) differ to those of Brazil and Shanghai, China (traffic accidents). In terms of gender, similar male:female distributions have been reported in Brazil^5^ (70.3% and 29.7%), Hebei province of China^3^ (71.1% and 28.9%), and Shanghai, China^6^ (67.8% and 32.2%). However, Elsoe *et al*.^4^ reported males accounted for 46.8% and females accounted for 53.2% of tibial plateau fractures in Denmark, and Schulak and Gunn^7^ reported no difference the male:female distribution in America. With respect to age, Elsoe *et al*.^4^ reported the highest frequency of fractures was observed in Danish patients aged between 40 and 60 years-old. In Brazil, Albuquerque *et al*.^5^ found the highest frequency was observed in the fifth decade. In America, Schulak and Gunn^7^ showed the highest frequency occurred between 45 and 60 years-old. In Hebei province of China, Zhang *et al*.^3^ observed the highest frequency in patients aged 41 to 50 years-old. However, in the classic 1979 study, Schatzker *et al*.^8^ reported tibial plateau fractures occurred most frequently in the sixth and seventh decades of life in Toronto, Canada. As for the lesion side, research by Albuquerque *et al*.^5^ showed right tibial plateau fractures accounted for 46.4% and left tibial plateau fractures accounted for 53.6% of cases in Brazil. In America, Schulak and Gunn^7^ found right- and left-sided tibial plateau fractures occurred in approximately equal numbers. In Shanghai, China, Luo *et al*.^6^ observed right and left tibial plateau fractures accounted for 58.2% and 41.8% of cases (not including bilateral tibial plateau fractures).

However, to the best of our knowledge, epidemiological studies of tibial plateau fractures related to the nature of employment and urban-rural differences have not yet been performed, and the epidemiological characteristics of tibial plateau fractures with respect to the Schatzker classification system^8^ and AO/OTA classification system^9^ have not yet been systematically investigated^3^,^4^,^5^,^6^. Besides that, China is still a developing country, and epidemiological trends can differ between developing countries and developed countries. At the end of 2015, China had a population of approximately 1.375 billion, accounting for about one-fifth of the world’s total population^10^, and the permanent population of Shaanxi province was over 37.93 million^11^. However, no systematic epidemiological study of tibial plateau fractures have been published for this large Chinese province. In addition, the most two commonly used classification systems are the Schatzker classification system^8^ and AO/OTA classification system^9^. As it is known to all, a number of tibial plateau fractures can be difficult to classify using either the Schatzker classification system or AO/OTA classification system, including horizontal shear of the entire plateau fractures^12^; subcondylar and bicondylar with coronal split fractures^13^; posterior, posteromedial and medial plateau shear-type fractures^14^; and tibial plateau fractures with cortical avulsion off the lateral rim or medial rim^15^,^16^. Although these types of fractures have been reported in the literature^4^,^5^,^6^,^14^,^17^, specific data was not provided. All of the above are the necessity of our study.

Based on the above background, the goal of our study was to investigate the Characteristics of unilateral tibial plateau fractures among hospitalized patients in Xijing Hospital (First Affiliated Hospital to the Fourth Military Medical University), which may reflect the characteristics of the disease in Shaanxi area somehow, to provide up-to-date information and references for prevention of this disease in the future. In addition, we aimed to evaluate the accuracy of the two existing classification systems for tibial plateau fractures.

## Materials and Methods

### Hospital and patients

Xijing Hospital (First Affiliated Hospital to the Fourth Military Medical University) is a Class III Grade I general hospital, the highest level hospital in China. All patients admitted to Xijing Hospital with unilateral tibial plateau fractures between September 2006 and August 2015 were included in this study. But in China, patients can choose the hospital according to their own wishes. Therefore, the patients in this study are not only came from Shaanxi province, but also included some patients from other regions of China.

### Inclusion and exclusion criteria

Inclusion criteria were hospitalized patients, age ≥15 years-old, and a diagnosis of unilateral tibial plateau fracture. The exclusion criteria were outpatients; age <15 years-old; presence of old, pathological or periprosthetic fractures, no or poor preoperative imaging; and bilateral tibial plateau fractures. Because the weight of patients with bilateral fractures is not the same as the weight of unilateral fractures patients. If we included patients with bilateral fractures, the results analysis will become complex and difficult to explain. Therefore, in order to ensure the homogeneity of the study, we only included patients with unilateral fractures, which accounts for the vast majority of tibial plateau fractures (about 97% of the total).

All fractures were classified according to the Schatzker classification system^8^ for tibial plateau fractures and the AO/OTA classification system^9^ for proximal tibia and fibula fractures. And according to the AO/OTA classification system, type 41-A fractures are extra-articular fractures; these fractures are not tibial plateau fractures and were not included in this study.

### Data collection

The imaging data and medical records of the patients, including preoperative plain radiographs, admission records, diagnosis, treatment procedures, nursing care and sociodemographic characteristics, were collected. These data were obtained using the Medical Image Management System and Hospital Information System at the Digital Centre of Xijing Hospital.

Data collection was conducted by one chief physician, one deputy chief physician and one attending physician from the Department of Orthopaedic Trauma. Training for the junior doctors led by the higher physician was scheduled before data collection and fracture classification. The attending physician assessed the fractures using the Schatzker classification system^8^ for tibial plateau fractures and the AO/OTA classification system^9^ for proximal tibia and fibula fractures. All imaging data included was classified by the attending physician, and these decisions were reviewed by the deputy chief physician.

Data were collected for 274 patients aged 15–85 years-old hospitalized with unilateral tibial plateau fractures. The variables of analyses included gender, age, side of lesion, type of employment, urban-rural residents, external cause, admission period, admission season, and fracture types according to the Schatzker classification and AO/OTA classification.

### Definitions and categories

(1) The patients were grouped according to age as follows: 15–29, 30–44, 45–59 and ≥60 years-old. (2) According to the Classification and codes of occupations^18^ (GB/T 6565–2015, P. R. China), the patients were grouped into three groups: office workers (Code: 10000–29900, 40400–40899, 41000–41099 and 41200–49900), physical labourers (Code: 30200–40399, 40900–40999, 41100–41199, 50000–69900 and 70000) and other types of occupation (including Code 80000, retirees, freelancers and unemployed persons). (3) The external cause of injury was classified using four types according to the ICD-10^19^ (International statistical Classification of Diseases and related health problems, Tenth Revision): traffic accidents (ICD-10 E Code: V01.- to V99.-), falls and indoor activity injuries (ICD-10 E Code: W00.- to W10.-, W18.- and W19.-), falls from height (ICD-10 E Code: W11.- to W17.-), and other external causes of injury, including contusion injuries or laceration injuries (ICD-10 E Code: W20.- to W31.-), sports injuries (ICD-10 E Code: X50.-) and fighting-induced injuries (ICD-10 E Code: W50.-). (4) All patients were grouped according to admission period as follows: September 2006 to August 2009, September 2009 to August 2012, and September 2012 to August 2015. (5) The patients were also grouped according to the season of admission (spring, summer, autumn, winter). (6) As mentioned above, a number of tibial plateau fractures were difficult to classify using either the Schatzker classification system^8^ or AO/OTA classification system^9^. In this study, these fractures were classified as difficult to classify fractures. Therefore, according to the Schatzker classification system, the fractures were classified as simple fractures (Types I, II, III, IV), complex fractures (Types V, VI), or difficult to classify fractures. Using the AO/OTA classification system, the fractures were classified as type 41-B fractures, type 41-C fractures, or difficult to classify fractures.

### Statistical analysis

Continuous variables are presented as the mean and standard deviation values; categorical data, as frequencies and percentages. The chi-square test was used to compare the external causes of injury between males and females, age groups, right- and left-sided injuries, types of employment, urban and rural residents, admission periods, admission seasons, and fracture types. SPSS 19.0 software (Statistical Package for Social Sciences, Inc., Chicago, IL, USA) was used for all analysis. *P* < 0.05 was considered statistically significant.

### Ethics statement

This study was conducted in according to the ethical principles of the Declaration of Helsinki^20^ and accordance with the ethical standards of the Ethics Committee of Xijing Hospital. The experimental protocols were approved by the Ethics Committee of Xijing Hospital. For the following reasons, the Ethics Committee of Xijing Hospital waived the need for written informed consent from the participants. Firstly, the Xijing Hospital is a teaching hospital, so the patient has been informed that the information may be used for scientific research in the “admission notice”. Secondly, in order to protect the patient’s privacy, the data were processed and analysed anonymously. Thirdly, we only collect the patient’s image data without using the human tissue, so it will not cause physical harm to the patients.

## Results

### General patient characteristics

Table 1 summarizes the frequency (n) and percentage (%) distributions for each characteristic assessed for the 274 adult patients hospitalized with unilateral tibial plateau fractures in Xijing Hospital.

### Analysis of external causes of tibial plateau fractures by demographic characteristics

As shown in Table 2, the external causes of tibial plateau fractures varied significantly between males and females (χ^2^ = 8.523, υ = 3, *P* = 0.036). Overall, traffic accident injuries, falls and indoor activity injuries, and falls from height injuries were the three most common external cause, accounting for 54.4%, 21.9%, and 13.1%, respectively. And the male to female ratios for them were 2.6:1, 1.4:1, and 5:1, respectively. With respect to other external cases, most of them were contusion injuries or lacerations, and fighting-induced injuries in males.

The external causes for each age group are summarized in Table 2. There was no significant difference in the distribution of external causes between different age groups (χ^2^ = 11.928, υ = 9, *P* = 0.217). The highest incidence of tibial plateau fractures occurred in the 30–59 years-old group (69.7%). Traffic accidents were the most common external cause for all age groups, followed by falls and indoor activity injuries. However, falls from height were the second most common external cause in the 30–44 years-old group. Other injuries were frequent in the group aged 30–59 years-old.

The frequency of each external cause for right- and left-sided tibial plateau fractures are listed in Table 2. There was no significant difference in the frequency of external causes between right- and left-sided tibial plateau fractures (χ^2^ = 2.094, υ = 3, *P* = 0.553). Right tibial plateau fractures accounted for 43.4% of cases and left tibial plateau fractures accounted for 56.6% of cases. Traffic accidents and falls and indoor activity injuries were the two most common external causes for both right and left tibial plateau fractures.

Significant differences in the external causes of tibial plateau fractures were observed among patients with different types of employment (Table 2; χ^2^ = 18.372, υ = 6, *P* = 0.005). The two leading external causes among office workers were traffic accidents (10.9%) and falls and indoor activity injuries (7.3%). The most common external causes among physical labourers were traffic accidents (36.1%) and falls from height (9.9%). Notably, traffic accidents were the most common external cause in every occupation group. Physical labourers were the most highly represented group among the cohort, accounting for 62.0% of all cases.

Overall, there were more rural residents (55.1%) than urban residents (44.9%) in this cohort. As shown in Table 2, there was a significant difference in the frequency of external causes between urban and rural residents (χ^2^ = 21.013, υ = 3, *P* < 0.001). Traffic accident injuries were the most common external cause among both urban (21.5%) and rural residents (32.8%). However, falls and indoor activity injuries were the second most common external cause among urban residents (15.3%), while falls from height were the second most common external cause among rural residents (8.0%).

### Analysis of external causes of tibial plateau fractures by admission period and admission season

The external causes of tibial plateau fractures for each three-year admission period are summarized in Table 3. There was no significant differences in the frequency of external causes between admission periods (χ^2^ = 4.327, υ = 6, *P* = 0.633). The number of patients treated in each three-year time period between 2006 and 2015 showed a gradually upward trend (18.2%, 31.0% and 50.7%, respectively). In all three time periods, traffic accidents and falls and indoor activity injuries were the two most common external causes.

There was no significant difference in the frequency of any external cause between seasons (Table 3, χ^2^ = 11.168, υ = 9, *P* = 0.264); traffic accidents and falls and indoor activity injuries were the two most common external causes throughout the year. Furthermore, analysis of seasonal variation showed an equal distribution of tibial plateau fractures throughout the year. From spring to winter, the distribution of patients hospitalized in each season was 27.7%, 22.6%, 24.5% and 25.2%, respectively.

### Analysis of external causes of tibial plateau fractures using the Schatzker classification system and AO/OTA classification system

Based on the Schatzker classification system, type II fractures were most common (30.7%), followed by type VI fractures (28.8%). Simple fractures (Types I, II, III, IV), complex fractures (Types V, VI), and difficult to classify fractures accounted for 56.9%, 31.4%, and 11.7% of tibial plateau fractures, respectively. There was a significant difference in the frequency of external causes between the Schatzker classification groups (Table 4; χ^2^ = 14.150, υ = 6, *P* = 0.028). Traffic accidents were the most common external cause in all groups. However, falls and indoor activity injuries were the second most common external cause in the simple fractures group, falls and indoor activity injuries and falls from height were the joint second most common external cause of complex fractures, and falls from height were the second most common external cause in the difficult to classify group.

Based on the AO/OTA classification system, type 41-B3 fractures were most common (37.2%), followed by type 41-C3 fractures (16.8%). Type 41-B fractures, type 41-C fractures, and the difficult to classify group accounted for 56.9%, 28.8%, and 14.2% of cases, respectively. The frequency of external causes varied significantly among the AO/OTA types (Table 4; χ^2^ = 16.291, υ = 6, *P* = 0.012). Traffic accidents were the most common external cause in all three groups. However, falls and indoor activity injuries were the second most common external cause of type 41-B fractures, and falls from height were the second most common external cause of type 41-C fractures and difficult to classify fractures.

In this series of 274 unilateral tibial plateau fractures among hospitalized adults, 11.7% (32 cases) of fractures were difficult to classify using the Schatzker classification system and 14.2% (39 cases) were difficult to classify using the AO/OTA classification system. All cases that were difficult to be classify using the Schatzker classification system were also difficult to classify using the AO/OTA classification system. In addition, all cases of Schatzker type V fractures were also difficult to classify using the AO/OTA classification system.

## Discussion

In this cohort of 274 hospitalized adult patients in Shaanxi, China, traffic accidents were the leading external cause of tibial plateau fractures (54.4%), followed by falls and indoor activity injuries (21.9%), and falls from height (13.1%). These results are in constrast to the cohorts studied by Elsoe *et al*.^4^ in Denmark, and slightly different to the cohort studied by Albuquerque *et al*.^5^ in Brazil and Luo *et al*.^6^ in Shanghai, China. Other external causes of injury, including industrial and agricultural-related contusion injuries or laceration injuries, sport-related injuries and fighting-induced injuries, accounted for 10.6% of cases in this cohort. However, other external causes of injury, including beach injuries, football related-injuries and fighting-induced injuries, accounted for 7.5% of tibial plateau fractures in the Brazilian cohort^5^.

Traffic accidents were the most common external cause of tibial plateau fractures in this cohort of Chinese patients. One primary reason is that China is a densely populated country with a high number of vehicles. At the end of 2015, motor vehicle ownership in China had reached 2.79 million^21^, ranked only second to the United States. In 2006, the country’s motor vehicle ownership was 1.45 million^21^ with a total of highway mileage of 3.46 million kilometres and road density of 36 km/100 square kilometres^22^. By the end of 2015, the total highway mileage had increased to 4.58 million kilometres and road density reached 48 km/100 square kilometres^22^. Another possible reason is that the low level of economic development in Shaanxi Province may contribute to the poor status of traffic safety facilities. For example, some drivers lack of adequate safety awareness and self-protection knowledge. These factors may explain the increase in numbers of casualties caused by traffic accidents observed in the present research. Therefore, we should pay more attention on improving the traffic safety facilities, promoting people’s self-awareness of public law or rules, enhancing people’s awareness of self-protection, and so on.

As for gender differences, males accounted for 71.5% and females accounted for 28.5% of cases in our study. Similar male:female distributions were reported by Albuquerque *et al*.^5^, Zhang *et al*.^3^ and Luo *at al.*^6^; however, Elsoe *et al*.^4^ and Schulak and Gunn^7^ observed different trends, Elsoe *et al*.^4^ observed a higher proportion of females than males, Schulak and Gunn^7^ observed no difference between genders (Table 5). In this cohort, the male:female ratios for traffic accidents and falls from height were 2.6:1 and 5:1, respectively, while the male:female ratio for falls and indoor activities was 1.4:1. This may be related to the first two types of injury are more common in construction, industrial and agricultural production, where men usually represent the vast majority of employees. With respect to other external causes, 18 cases were related to contusion injuries or laceration injuries: 15 in males and three in females. We believe this trend is related to the fact males are more commonly engaged in industrial production and operation of agricultural machinery. It also maybe related to that, in rural areas of China, males usually work to support their family whereas females often stay at home to do housework. So many more males than females work in construction, transportation, industrial and agricultural production, and other high intensity labours. For this part, our data indicates that males were most likely to suffer from unilateral tibial plateau fractures than females. The aetiology of tibial plateau fractures varies between countries and even from one region to another within the same country, and is influenced by socioeconomic development, productivity and mode of production, as well as cultural and environmental factors. So, we should pay more attention to male labourers to reduce the occurrence of tibial plateau fractures.

The highest frequency of tibial plateau fractures occurred in the 30–59 years-old group (69.7%), in agreement with Elsoe *et al*.^4^, Albuquerque *et al*.^5^, Schulak and Gunn^7^ and Zhang *et al*.^3^ (Table 5). However, we observed no significant difference in the frequency of external causes between different age groups in this cohort. Traffic accidents were the most common external cause in every age group. Falls from height were the second most common external cause in the 30–44 years-old group; however, falls from height were significantly less common in the groups aged 15–29, 45–59 and ≥60 years-old. This may be related to the fact individuals aged 30–44 are more likely to be engaged in construction, industrial and agricultural production, and are therefore more prone to suffer from falls from height. While Schatzker *et al*.^8^ reported tibial plateau fractures occurred most frequently in the sixth and seventh decades of life in a study performed in 1975, we believe that the increase in the number of traffic accidents over time may have greatly reduced the average age of the patients. In this part, we found that the 30–59 years-old group people were more likely to suffer from unilateral tibial plateau fractures. Therefore, we should pay more attention to 30–59 years-old labourers to reduce the occurrence of tibial plateau fractures.

Right tibial plateau fractures accounted for 43.4% of cases and left tibial plateau fractures accounted for 56.6% of cases in this cohort. The results of the study by Albuquerque *et al*.^5^ in Brazil are generally in agreement with our data. However, Schulak and Gunn^7^ and Luo *et al*.^6^ reported different trends in America and Shanghai, China, as described in the introduction (Table 5). Further analysis is required to explore if these variations are true, and if so, explore the factors that affect the incidence of right and left tibial plateau fractures.

With regard to the relationships between external causes of injury and type of employment, traffic accidents were the most common external cause in this cohort. Patients injured by any external cause were mainly physical labourers. The second most common external cause in physical labourers was falls from height, whereas falls and indoor activity injuries were the second most common external causes in office workers and people with other types of employment. These trends may reflect differences in the working environments. Compared to office workers, physical labourers are more likely to suffer falls from height. Therefore, in order to to reduce the occurrence of tibial plateau fractures, physical labourers should pay more attention to traffic accidents and falls from height injuries, whereas office workers should pay more attention to traffic accidents, falls and indoor activity injuries.

In terms of urban-rural differences, no specific data is available on the relationship between the external causes of tibial plateau fractures and the patients’ area of residence in previous studies. Among both urban and rural residents, traffic accidents were the most common external cause. Fractures caused by all external causes (except fall and indoor activity injuries) were mainly observed in rural residents. The second most common external cause among urban residents was falls and indoor activity injuries. However, the second most common external cause in rural residents was falls from height. This observation may be related to the presence of migrant workers that travel from rural to urban areas, and who may be more likely to suffer fall from height injuries at work, such as construction and outdoor production activities, which is a common phenomenon in China at present. Therefore, as previously stated, paid more attention to rural residents, especially those who work at heights in urban areas, may help to reduce the occurrence of tibial plateau fractures.

Data from previous epidemiological studies on the classification of tibial plateau fractures is summarized in Table 5. Albuquerque *et al*.^5^ reported simple (Schatzker type I, II, III and IV) and complex fractures (type V and VI) accounted for 64.0% and 36.0% of cases in Brazil, respectively; Schatzker type II fractures were most common (35.1%), followed by type VI fractures (20.1%). Based on the AO/OTA classification system, type 41-B and 41-C fractures accounted for 64.9% and 35.1% of cases in Brazil, respectively; type 41-B3 fractures were most common (36.9%), followed by type 41-C3 fractures (21.3%). Similarly, Elsoe *et al*.^4^ showed AO/OTA type 41-B and type 41-C fractures accounted for 65.1% and 34.9% of tibial plateau fractures in Denmark; type 41-B3 fractures were most common (38.3%), followed by type 41-C3 fractures (19.0%). These data are in agreement with the results of the present study. Zhang *et al*.^3^ reported simple fractures (type I, II, III and IV) and complex fractures (type V and VI) accounted for 69.1% and 30.9% of cases in Hebei, China; type VI fractures were most common (22.3%), followed by type II and III fractures (both 18.3%). Based on the AO/OTA classification system, type 41-B and 41-C fractures accounted for 67.9% and 32.1%, respectively; our results do not completely agree with the data reported by Zhang *et al*.^3^. Moreover, Luo *et al*.^6^ reported simple (type I, II, III, IV), complex (type V and VI) and difficult to classify fractures accounted for 75.2%, 20.7% and 4.0% of fractures in Shanghai, China, respectively; Schatzker type II fractures were most common (39.9%), followed by type IV fractures (26.9%). The results of our study were significantly different (χ^2^ = 25.502, υ = 2, *P* < 0.001). A number of reasons may explain these differences between studies. Firstly, both unilateral and bilateral tibial plateau fractures were included in all of the previous studies^3^,^4^,^5^,^6^. Whereas, in order to ensure the homogeneity of the study, we only included patients with unilateral tibial plateau fractures in our study (bilateral fractures only accounted for about 3%). Secondly, we classified some fractures as difficult to classify, as they did not conform to the classical Schatzker or AO/OTA classification systems; this method was not adopted in previous studies^3^,^4^,^5^. Forcing each fracture to be classified using the Schatzker or AO/OTA classification system must alter the proportions of each type of fracture. Thirdly, fracture types generally reflect the mechanism of injury and violent energy. Working environments vary from one country to another and even from one region to another within the same country, and are influenced by socioeconomic development, cultural and environmental factors, which may further affect the epidemiology of fractures, and fracture types.

This study has some limitations. Firstly, China has not yet implemented a strict grading system of diagnosis-treatment and two-way referral system, so patients are free to choose the hospital they attend according to their personal wishes. Therefore, the patients in this cohort not only came from Shaanxi province (72.3%), but also other regions of China (27.7%). In addition, this was a single-centre study. Therefore, it is difficult to provide an accurate analysis of the incidence of tibial plateau fractures. We believe implementation of the diagnosis-treatment grading system and two-way referral system in China will help to relatively “fix” the geographical location of patients and gradually standardize the medical treatment process. Further research is required to provide a more comprehensive and objective understanding of the epidemiology of tibial plateau fractures in China, and may provide more objective, scientific data for the formulation of national health policy.

This analysis of patients hospitalized at a single orthopaedic trauma centre in China demonstrates rural male physical labourers aged between 30–59 years-old are the group most likely to suffer unilateral tibial plateau fractures, with traffic accidents, falls and indoor activity injuries, or falls from height the main external causes. Overall, Schatzker type II fractures and AO/OTA type 41-B3 fractures were most common types. The external causes varied between gender, type of employment, urban-rural residents and both the Schatzker and AO/OTA classifications. However, the external causes did not differ significantly among age groups, admission periods, admission seasons, and right- and left-sided injuries. We should pay more attention to the related people and professions, which contributed to the high occurrence of tibial plateau fractures in this cohort. In addition, a number of fractures (about 11% to 15%) were difficult to classify using the Schatzker classification system or AO/OTA classification system. Therefore, further improvements are required for both Schatzker classification system and AO/OTA classification system to tibial plateau fractures.

## Additional Information

**How to cite this article**: Liu, Y. *et al*. Characteristics of unilateral tibial plateau fractures among adult patients hospitalized at an orthopaedic trauma centre in China. *Sci. Rep.* **7**, 40647; doi: 10.1038/srep40647 (2017).

**Publisher's note:** Springer Nature remains neutral with regard to jurisdictional claims in published maps and institutional affiliations.

## Figures and Tables

**Table 1:** Characteristics of the 274 adult patients hospitalized with unilateral tibial plateau fractures.

Variable	Category	*n*	%
Gender	Male	196	71.5
	Female	78	28.5
Age (years)	43.10 ± 13.55	(15–85)	
Side of lesion	Right	119	43.4
	Left	155	56.6
Nature of employment	Office worker	63	23.0
	Physical labourer	170	62.0
	Other	41	15.0
Area of residence	Urban	123	44.9
	Rural	151	55.1
Admission period	Sept 2006 to Aug 2009	50	18.2
	Sept 2009 to Aug 2012	85	31.0
	Sept 2012 to Aug 2015	139	50.7
Admission season	Spring	76	27.7
	Summer	62	22.6
	Autumn	67	24.5
Winter	69	25.2	
Schatzker classification	Type I	24	8.8
	Type II	84	30.7
	Type III	12	4.4
	Type IV	36	13.1
	Type V	7	2.6
	Type VI	79	28.8
	Other	32	11.7
AO/OTA classification	41-B1	39	14.2
	41-B2	15	5.5
	41-B3	102	37.2
	41-C1	19	6.9
	41-C2	14	5.1
	41-C3	46	16.8
	Other	39	14.2
External cause of injury	Traffic accidents	149	54.4
	Falls and indoor activity injuries	60	21.9
	Falls from height	36	13.1
	Other	29	10.6

**Table 2:** Frequency of different external causes of tibial plateau fractures [*n* (%)] by gender, age, side of lesion, type of employment and area of residence.

Variable	Group	Traffic accidents	Falls and indoor activity injuries	Falls from height	Other	Total
Gender^*a^	Males	108 (39.4)	35 (12.8)	30 (10.9)	23 (8.4)	196 (71.5)
	Females	41 (15.0)	25 (9.1)	6 (2.2)	6 (2.2)	78 (28.5)
	Total	149 (54.4)	60 (21.9)	36 (13.1)	29 (10.6)	274 (100.0)
Age (years)^b^	15–29	32 (11.7)	7 (2.6)	6 (2.2)	5 (1.8)	50 (18.2)
	30–44	48 (17.5)	17 (6.2)	19 (6.9)	11 (4.0)	95 (34.7)
	45–59	52 (19.0)	25 (9.1)	9 (3.3)	10 (3.6)	96 (35.0)
	≥60	17 (6.2)	11 (4.0)	2 (0.7)	3 (1.1)	33 (12.0)
	Total	149 (54.4)	60 (21.9)	36 (13.1)	29 (10.6)	274 (100.0)
Side of lesion^c^	Right	63 (23.0)	30 (10.9)	16 (5.8)	10 (3.6)	119 (43.4)
	Left	86 (31.4)	30 (10.9)	20 (7.3)	19 (6.9)	155 (56.6)
	Total	149 (54.4)	60 (21.9)	36 (13.1)	29 (10.6)	274 (100.0)
Type of employment^*d^	Office workers	30 (10.9)	20 (7.3)	6 (2.2)	7 (2.6)	63 (23.0)
	Physical labourers	99 (36.1)	24 (8.8)	27 (9.9)	20 (7.3)	170 (62.0)
	Other	20 (7.3)	16 (5.8)	3 (1.1)	2 (0.7)	41 (15.0)
	Total	149 (54.4)	60 (21.9)	36 (13.1)	29 (10.6)	274 (100.0)
Area of residence^*e^	Urban	59 (21.5)	42 (15.3)	14 (5.1)	8 (2.9)	123 (44.9)
	Rural	90 (32.8)	18 (6.6)	22 (8.0)	21 (7.7)	151 (55.1)
	Total	149 (54.4)	60 (21.9)	36 (13.1)	29 (10.6)	274 (100.0)

Note: a (χ^2^ = 8.523, υ = 3, *P* = 0.036); b (χ^2^ = 11.928, υ = 9, *P* = 0.217); c (χ^2^ = 2.094, υ = 3, *P* = 0.553); d (χ^2^ = 18.372, υ = 6, *P* = 0.005); e (χ^2^ = 21.013, υ = 3, *P* < 0.001).

**Table 3:** Frequency of different external causes of tibial plateau fractures [*n* (%)] by admission period and admission season.

Variable	Group	Traffic accidents	Falls and indoor activity injuries	Falls from height	Other	Total
Admission period^a^	Sept 2006 to Aug 2009	23 (8.4)	11 (4.0)	9 (3.3)	7 (2.6)	50 (18.2)
	Sept 2009 to Aug 2012	49 (17.9)	21 (7.7)	9 (3.3)	6 (2.2)	85 (31.0)
	Sept 2012 to Aug 2015	77 (28.1)	28 (10.2)	18 (6.6)	16 (5.8)	139 (50.7)
	Total	149 (54.4)	60 (21.9)	36 (13.1)	29 (10.6)	274 (100.0)
Admission season^b^	Spring	42 (15.3)	15 (5.5)	8 (2.9)	11 (4.0)	76 (27.7)
	Summer	27 (9.9)	20 (7.3)	8 (2.9)	7 (2.6)	62 (22.6)
	Autumn	36 (13.1)	12 (4.4)	11 (4.0)	8 (2.9)	67 (24.5)
	Winter	44 (16.1)	13 (4.7)	9 (3.3)	3 (1.1)	69 (25.2)
	Total	149 (54.4)	60 (21.9)	36 (13.1)	29 (10.6)	274 (100.0)

Note: a (χ^2^ = 4.327, υ = 6, *P* = 0.633); b (χ^2^ = 11.168, υ = 9, *P* = 0.264).

**Table 4:** Frequency of different external causes of tibial plateau fractures [*n* (%)] by Schatzker classification groups and AO/OTA classification groups.

Variable	Group	Traffic accidents	Falls and indoor activity injuries	Falls from height	Other	Total
Schatzker classification^a^	Simple fracture	88 (32.1)	41 (15.0)	14 (5.1)	13 (4.7)	156 (56.9)
	Complex fracture	48 (17.5)	13 (4.7)	13 (4.7)	12 (4.4)	86 (31.4)
	Other	13 (4.7)	6 (2.2)	9 (3.3)	4 (1.5)	32 (11.7)
	Total	149 (54.4)	60 (21.9)	36 (13.1)	29 (10.6)	274 (100.0)
AO/OTA classification^b^	41-B fracture	88 (32.1)	41 (15.0)	14 (5.1)	13 (4.7)	156 (56.9)
	41-C fracture	47 (17.2)	10 (3.6)	12 (4.4)	10 (3.6)	79 (28.8)
	Other	14 (5.1)	9 (3.3)	10 (3.6)	6 (2.2)	39 (14.2)
	Total	149 (54.4)	60 (21.9)	36 (13.1)	29 (10.6)	274 (100.0)

Note: a (χ^2^ = 14.150, υ = 6, *P* = 0.028); b (χ^2^ = 16.291, υ = 6, *P* = 0.012).

**Table 5:** Characteristics of tibial plateau fractures in different countries and regions (%).

Variable	Group	Brazil	Denmark	Hebei China	Shanghai China	Shaanxi China
Ratio of gender	Male:Female	2.37:1	0.88:1	2.46:1	2.08:1	2.52:1
Ratio of lesion side	Right:Left	0.87:1	—	—	1.39:1	0.77:1
Most common age	year	50–60	40-60	41–50	—	30-59
Schatzker classification	Simple fracture	64.0	—	69.1	75.2	56.9
	Complex fracture	36.0	—	30.9	20.7	31.4
	Other	—	—	—	4.0	11.7
	First common	II (35.1)	—	VI (22.3)	II (39.9)	II (30.7)
	Second common	VI (20.1)	—	II and III (both 18.3)	IV (26.9)	VI (28.8)
AO/OTA classification	41-B fracture	64.9	65.1	67.9	—	56.9
	41-C fracture	35.1	34.9	32.1	—	28.8
	Other	—	—	—	—	14.2
	First common	41-B3 (36.9)	41-B3 (38.3)	—	—	41-B3 (37.2)
	Second common	41-C3 (21.3)	41-C3 (19.0)	—	—	41-C3 (16.8)

Note: “–”indicates the data could not be extracted from the corresponding study.
